# Anopheles gambiae densovirus (AgDNV) negatively affects Mayaro virus infection in *Anopheles gambiae* cells and mosquitoes

**DOI:** 10.1186/s13071-020-04072-8

**Published:** 2020-04-22

**Authors:** Nadya Urakova, Marco Brustolin, Renuka E. Joseph, Rebecca M. Johnson, Sujit Pujhari, Jason L. Rasgon

**Affiliations:** 1 Department of Entomology, The Pennsylvania State University, University Park, PA USA; 2 The Center for Infectious Disease Dynamics, The Pennsylvania State University, University Park, PA USA; 3 The Huck Institutes of the Life Sciences, The Pennsylvania State University, University Park, PA USA

**Keywords:** *Anopheles gambiae*, Densovirus, Mayaro virus, Cell culture, Vector competence

## Abstract

**Background:**

Recent studies demonstrate that insect-specific viruses can influence the ability of their mosquito hosts to become infected with and transmit arboviruses of medical and veterinary importance. The aim of this study was to evaluate the interactions between Anopheles gambiae densovirus (AgDNV) (*Parvoviridae*) (a benign insect-specific virus that infects *An. gambiae* mosquitoes) and Mayaro virus (MAYV) (*Togaviridae*) (an emerging human pathogen that can be transmitted by *An. gambiae*) in both insect cell culture and mosquitoes.

**Methods:**

For *in vitr*o studies, *An. gambiae* Mos55 cells infected or uninfected with AgDNV were infected with MAYV. For *in vivo* studies, *An. gambiae* mosquitoes were injected intrathoracically with AgDNV and 4 days later orally infected with MAYV. Mosquitoes were dissected 10 days after MAYV infection, and MAYV titers in the body, legs and saliva samples quantified using focus-forming assay.

**Results:**

MAYV virus replication was reduced 10–100-fold in *An.* *gambiae* Mos55 cells infected with AgDNV. In mosquitoes, there was a significant negative correlation between AgDNV and MAYV body titers 10 days post-blood meal.

**Conclusions:**

AgDNV infection was associated with reduced production of MAYV in cell culture, and reduced body titers of MAYV in *An. gambiae* mosquitoes. As densovirus infections are common in natural mosquito populations, these data suggest that they may affect the epidemiology of viruses of medical importance.
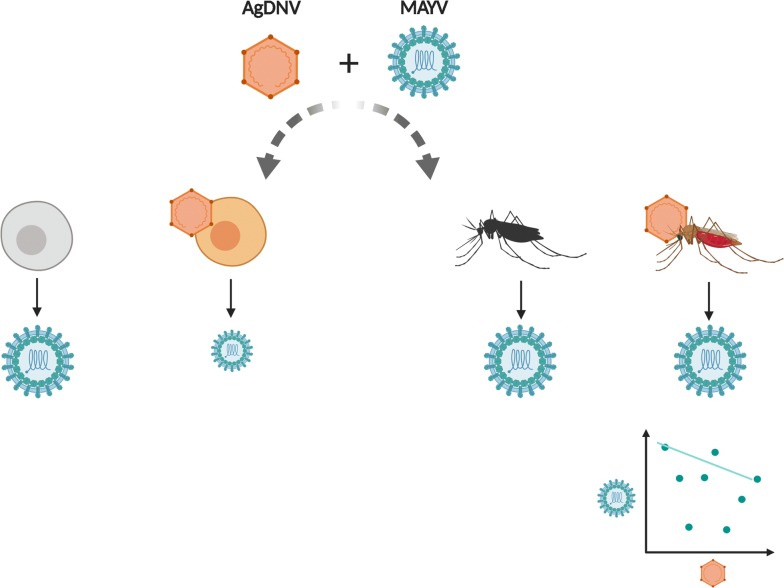

## Background

Insect-specific viruses (ISVs) are unable to infect vertebrate cells but can efficiently replicate in insects such as mosquitoes. As mosquitoes can also be infected with and transmit arboviruses of medical and veterinary importance, ISVs are being considered as potential biocontrol tools for mosquitoes.

Several studies have shown that many widely distributed mosquito cell lines which are used as models to study arbovirus infections in mosquito vectors, such as U4.4 (*Aedes albopictus*), Aag2, CCL-125 (*Aedes aegypti*) and HSU (*Culex quinquefasciatus*), are persistently infected with multiple ISVs that do not cause cytopathic effect or affect cell growth, and thus remain unnoticed [1, 2]. Isolation of arboviruses from clinical samples and virus production for vector competence studies often employ the use of mosquito cells that can be infected with ISVs [3]. For example, the two widely used Zika virus stocks produced in C6/36 (*Ae. albopictus*) cells were contaminated with mosquito densoviruses [3]. It is unknown whether blood-feeding using arbovirus stocks contaminated with insect-specific viruses can result in the establishment of mixed infections.

Although vertical transmission, when the virus is passed transovarially from infected female mosquitoes to their progeny, is believed to be the main mechanism of maintenance of ISVs in mosquito populations [4], it is possible that ISVs can be transmitted *via* other mechanisms. It was shown that the insect-specific alphavirus Eilat virus could infect *Ae. aegypti* mosquitoes *via* blood-feeding and disseminate from the midgut. Surprisingly, Eilat virus replication could not be detected in the ovaries after intrathoracic injections of the virus, while the salivary glands were readily infected [4]. Given the fact that mosquitoes can feed on hemolymph of other insects such as caterpillars [5], it is possible that mosquitoes can be infected with ISVs *via* oral route as well. However, studies into the effects of ISVs on arbovirus infections are still in an early stage and more research is needed to better understand how ISVs can influence arbovirus replication in cell culture and affect vector competence estimates [6].

Anopheles gambiae densonucleosis virus (AgDNV) is a non-enveloped single-stranded insect-specific DNA virus in the family *Parvoviridae* that has restricted host range and infects anopheline mosquitoes [7]. Unlike most mosquito densoviruses, AgDNV does not cause significant pathological effects in its mosquito host [8].

Although *An. gambiae* mosquitoes are usually considered as the major vector of human malaria, recent evidence demonstrated that this species can also transmit Mayaro virus (MAYV) [9], an emerging mosquito-borne alphavirus currently circulating in South America and the Caribbean and with potential to spread to other regions including Europe and the USA [9, 10]. MAYV is an enveloped single-stranded positive-sense RNA virus from the family *Togaviridae*. Based on phylogenetic studies, MAYV strains are classified into two major genotypes, genotype D (widely dispersed) and L (limited), and a third minor genotype, N (new) [10, 11]. In this study, we used AgDNV, MAYV, and *An. gambiae* mosquitoes and cells as a model system to study an effect of a benign ISV on arbovirus replication.

## Methods

### Mosquito rearing

*Anopheles gambiae* mosquitoes (Keele strain) were originally obtained from The National Institutes of Health (Bethesda, MD, USA). Mosquitoes were reared at the PSU Millennium Sciences Complex insectary in 30 × 30 × 30 cm cages under the following environmental conditions: 27 ± 1 °C; 12:12 hours light:dark diurnal cycle; 80% relative humidity. For reproduction, mosquitoes were maintained on expired anonymous human blood using a membrane feeder as previously described [9]. Larvae were fed on ground fish flakes (TetraMin). Adult mosquitoes were provided with 10% sucrose solution as a carbohydrate source.

### Cells

*Anopheles gambiae* Mos55 and Sua5B cells were grown in Schneider’s media (Gibco/Thermo Fisher Scientific, Waltham MA, USA) supplemented with 10% fetal bovine serum (FBS) (Gibco/Thermo Fisher Scientific), 100 µg/ml of streptomycin (Gibco/Thermo Fisher Scientific) and 100 units/ml of penicillin (Gibco/Thermo Fisher Scientific) at 28 °C. African green monkey kidney (Vero, ATCC CCL-81) cells (ATCC, Manassas, Virginia, USA) were grown in Dulbecco’s modified Eagle’s medium (DMEM) (Gibco/Thermo Fisher Scientific) supplemented with 10% FBS (Gibco/Thermo Fisher Scientific), 100 µg/ml of streptomycin (Gibco/Thermo Fisher Scientific) and 100 units/ml of penicillin (Gibco/Thermo Fisher Scientific) at 37 °C in 5% CO_2._

### Viruses

AgDNV (GenBank: EU233812.1) stocks were prepared from Sua5B cells which are persistently infected with this virus [12]. Cells were lysed by vortexing with sterile 3 mm borosilicate glass beads for 5 min and centrifuged for 10 min at 12,000×*g* at 4 °C in a benchtop microcentrifuge to remove cell debris. Cleared lysates were used for infections and injections or stored at − 80 °C.

MAYV strains BE AN343102 and BE AR505411 were obtained from BEI Resources (Manassas, VA, USA). BE AN343102 is a genotype D strain originally isolated from a monkey in Para (Brazil) in May 1978. BE AR505411 is a genotype L strain originally isolated from *Haemagogus janthinomys* mosquitoes in Para (Brazil) in March 1991. To produce viral stocks, viruses were propagated on Vero cells and stored at − 80 °C. MAYV stocks were quantified using focus-forming assay (see below).

### qPCR for AgDNV quantification

Total DNA was extracted from 100 µl of AgDNV stocks or mosquito homogenates using E.Z.N.A. Tissue DNA Kit (Omega Bio-Tek, NorCross, GA, USA) kit according to the manufacturer’s instructions. qPCR was performed using PerfeCTa SYBR Green FastMix (Quantabio, Beverly, MA, USA) on a Rotor-Gene Q qPCR machine (Qiagen, Hilden, Germany) using the following primers: 5′-GGC ATC AAT GTG GGA CCA AG-3′ (forward) and 5′-CCG TTA GCA AGC GTT GTC TG-3′ (reverse), and thermocycling conditions: 95 °C for 2 min for initial denaturation; 40 cycles at 95 °C for 10 s, 60 °C for 40 s, 72 °C for 30 s for DNA amplification and data acquisition; 55–99 °C (5 s per increment) for the melt curve analysis. A standard curve was generated using a dilution series of a plasmid encoding AgDNV genome as previously described [12].

### Antibodies

Mouse monoclonal anti-chikungunya virus E2 envelope glycoprotein antibodies, clone CHK-48 (produced *in vitro*) (NR-44002) were obtained from BEI Resources, NIAID, NIH. These antibodies were shown to be cross-reactive with MAYV antigens of both BE AN343102 and BE AR505411 strains. Goat anti-mouse IgG (H+L) highly cross-adsorbed secondary antibodies, Alexa Fluor 488 (A-11029) were purchased from Invitrogen. Both antibodies were used in focus-forming assays at a dilution of 1:1000.

### Focus-forming assay for MAYV quantification

Vero cells were seeded in 96-well plates at a density of 3 × 10^4^ cells/well. Ten-fold serial dilutions of virus samples in serum-free DMEM were made in 96-well plates and 30 µl of prepared solutions per well were used for infections. Cells were infected at 37 °C for 2 hours, infectious solutions removed, and cells covered with 100 µl of DMEM containing 1% methylcellulose (Sigma-Aldrich, St. Louis, MO, USA). After 24 hours, overlay medium was removed, cells were fixed with 4% paraformaldehyde (Sigma-Aldrich) in phosphate-buffered saline (PBS) (Gibco/Thermo Fisher Scientific) for 15 min and permeabilized and blocked using a blocking solution containing 3% bovine serum albumin (Sigma-Aldrich) and 0.125% TritonX in PBS for 30 min. Primary and secondary antibodies were diluted in blocking solution and incubated for 2 hours and 1 hour, respectively. All incubations and washing steps using PBS were carried out at room temperature. After the final wash, cells were dried briefly to remove the excess of liquid, and MAYV foci immediately counted using an Olympus BX41 inverted microscope equipped with an UPlanFI 4X objective and a FITC filter.

### Mixed AgDNV and Mayaro virus infections in cell culture

Mos55 cells were seeded in 6-well plates, infected with AgDNV using 100 µl of AgDNV stock obtained from Sua5B cells (~10^11^ genome equivalents (GE)/ml) in serum-free Schneider’s medium or mock-infected, incubated for 4 days to establish AgDNV infection and superinfected with MAYV at an MOI ≤ 0.01. Medium samples were collected at 0, 4, 8, 12, 24, 48, 72 and 120 hours post-superinfection and MAYV titers were evaluated using focus-forming assays. Cells were infected in triplicates using two different MAYV strains (BE AN and BE AR), and the experiment was performed twice.

### Mosquito injections with AgDNV

Two to three-day-old *An.* *gambiae* female mosquitoes were infected with AgDNV *via* intrathoracic injection. Mosquitoes were anesthetized on ice and injected with 69 nl of AgDNV stock (10^11^ GE/ml) or complete Schneider’s medium as a control, using a Nanoject II injector (Drummond Scientific, Broomall, PA, USA).

### Vector competence assays

Four days after injection with AgDNV, *An. gambiae* female mosquitoes were fed for 30–45 min on infected human blood spiked with 10^7^ infectious MAYV (BE AN343102 strain) particles per ml. After feeding, mosquitoes were anesthetized on ice, fully engorged females selected and placed in cardboard cages. Ten days after blood-feeding, survived mosquitoes were anesthetized with triethylamine (Sigma-Aldrich) and processed for vector competence assays. To collect saliva samples, mosquitoes were forced to salivate in glass capillaries filled with a mix of 50% sucrose solution and FBS (1:1) for 30 min. Body, legs and saliva samples were collected in mosquito diluent containing 20% of FBS, 100 µg/ml of streptomycin, 100 units/ml of penicillin, 50 µg/ml gentamicin, and 2.5 µg/ml Amphotericin B in PBS. Body and legs samples were homogenized by a single zinc-plated, steel, 4.5 mm bead using TissueLyser II (Qiagen) at 30 Hz for 2 min and centrifuged at 4000× *rpm* at 4 °C for 5 min in a bench top centrifuge to clear the homogenates. Samples were stored at − 80 °C. 20 µl of body and legs samples were used to prepare 10-fold serial dilutions as described above, and 30 µl of undiluted saliva samples in mosquito diluent were used in focus-forming assay.

### Data analysis

Infection frequency data between treatments were analyzed using Fisher’s exact text. To determine relationships between AgDNV and MAYV body titer, the Spearman’s rank correlation test was used, as assumptions for Pearson’s correlation were violated. All statistical analyses were performed in GraphPad Prism version 7 for Windows (GraphPad Software, San Diego, CA).

## Results

### AgDNV infection reduced MAYV titers in *An. gambiae* Mos55 cells

**Fig. 1:**
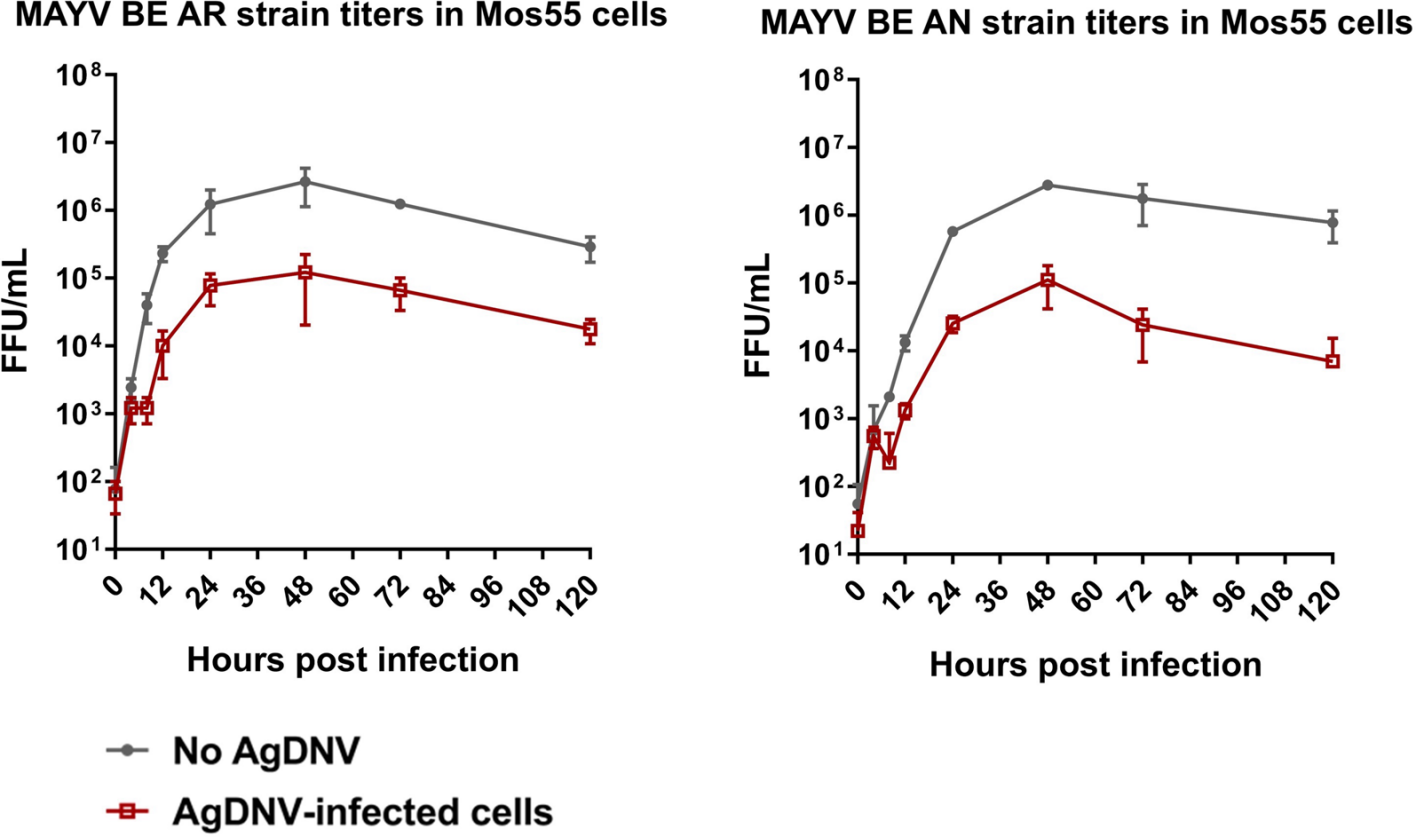
MAYV replication in AgDNV-infected or uninfected Mos55 cells: MAYV BE AR strain and MAYV BE AN strain

Two different MAYV strains (BE AR and BE AN) were tested in cell culture experiments. Results showed that MAYV titers for both strains were 10–100-fold lower when grown on AgDNV-infected Mos55 cells compared to uninfected cells at all time points tested (Fig. 1). The titers of MAYV propagated on both AgDNV-infected and uninfected Mos55 cells peaked at 48 hours post-MAYV infection and then gradually decreased between 48 hours and 120 hours post-infection.

### Negative correlation between AgDNV and MAYV titers in mosquitoes

**Fig. 2:**
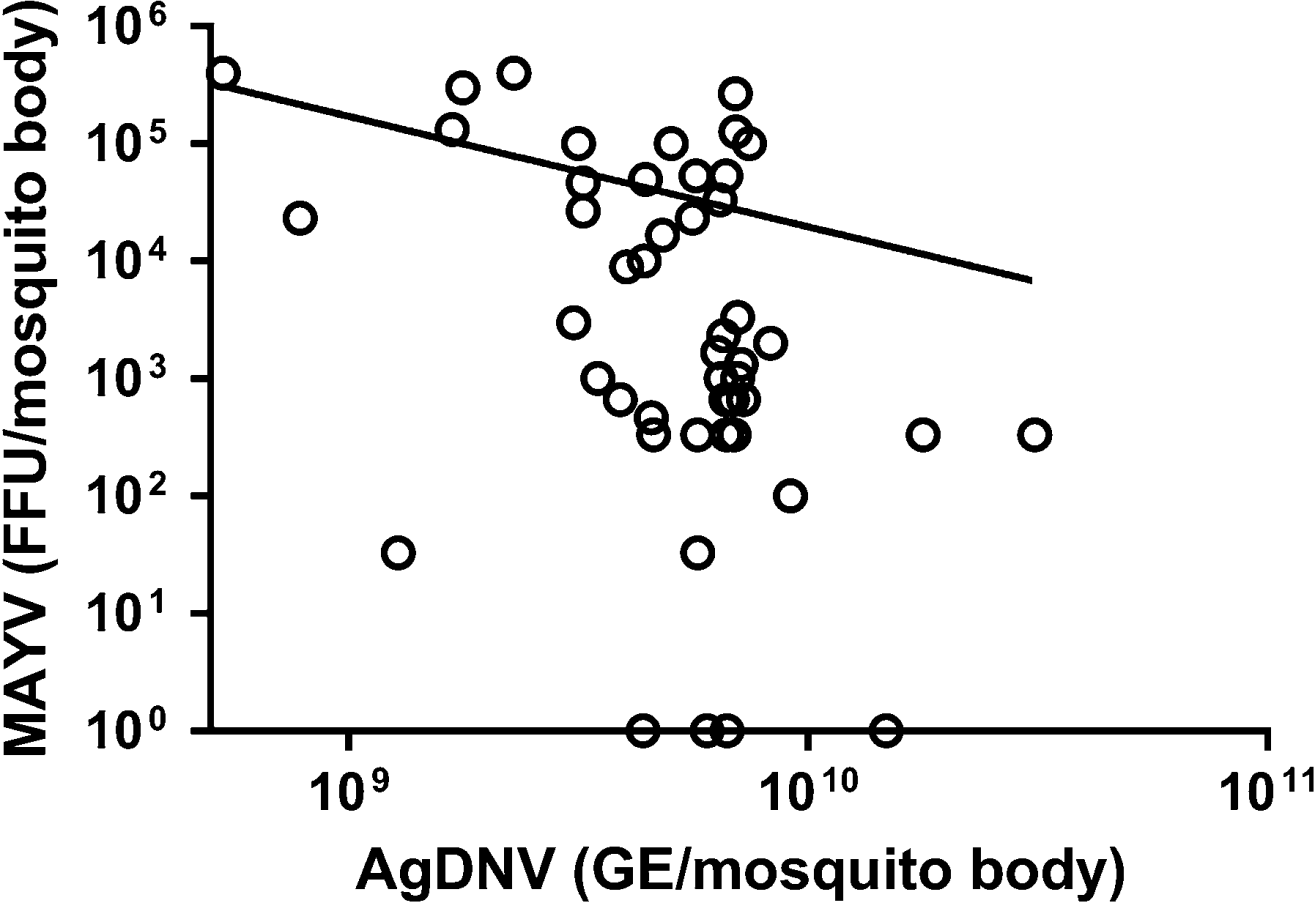
Correlation between MAYV (BE AN strain) body titers and AgDNV titers in *An. gambiae*. MAYV and AgDNV body titers for each mosquito were plotted and analysed with the Spearman’s rank correlation test to determine relationships. There was a significant negative correlation between MAYV and AgDNV body titers 10 days post-blood meal (*n* = 48, *r*_*s*_= − 0.365, *P* = 0.0117)

Replication of AgDNV in injected mosquitoes was confirmed by qPCR (Additional file 1: Figure S1). In AgDNV-injected mosquitoes, AgDNV titers (measured as AgDNV GE/mosquito) were ~1000-fold higher compared to the inoculum used for injections. Randomly selected body samples from medium-injected mosquitoes were tested negative for AgDNV DNA. In mosquito body samples, we observed a significant negative correlation between AgDNV and MAYV titers (*n* = 48, *r*_*s*_ = − 0.365, *P* = 0.0117) (Fig. 2). Infection, dissemination, and transmission rates did not differ significantly between treatments (Table 1).

**Table 1:** Infection, dissemination and transmission rates for MAYV in *An. gambiae* mosquitoes infected or uninfected with AgDNV at day 10 post-MAYV infection

Group	Infection rate	Dissemination rate	Transmission efficiency
Experiment 1			
MAYV only	100% (13/13)	15% (2/13)	0
AgDNV+MAYV	79% (11/14)	54% (6/11)	0
Experiment 2			
MAYV only	93% (28/30)	50% (14/28)	0
AgDNV+MAYV	97% (33/34)	48% (16/33)	6% (2/34)

*Notes*: Infection rate: percentage of engorged mosquitoes with infected body; dissemination rate: percentage of mosquitoes with infected bodies that were also tested positive for infection in the legs; transmission efficiency: percentage of engorged mosquitoes with infectious saliva

## Discussion

It was previously shown that *Ae. albopictus* densovirus reduced production of dengue virus (serotype 2) in both cell culture (C6/36 cells) and *Ae. albopictus* mosquitoes [13, 14], while it had no effect on chikungunya virus RNA replication in C6/36 cells and *Ae. aegypti* mosquitoes [15]. In contrast to other densoviruses, which can be pathogenic to mosquito larvae and mosquito cells in culture [16], AgDNV is relatively benign and does not cause major pathological signs in mosquitoes or cells [8]. Although *An. gambiae* mosquitoes are usually considered as a vector of malaria rather than arboviruses, recent work demonstrated that the potential for *An. gambiae* (and other *Anopheles* species) to transmit medically important viruses, such as MAYV, is underestimated [9]. In this study, we showed that AgDNV infections significantly reduced MAYV titers in cell culture and in mosquitoes *in vivo*. We collected saliva at day 10 post-MAYV infection and the transmission efficiency appeared lower at this time point. Further studies that aim specifically to evaluate the effects of AgDNV on MAYV transmission at later time points should be conducted to gain a more complete understanding of the transmission dynamics in this co-infection system, as transmission dynamics can change over the course of the mosquito lifespan.

As recent studies suggest that ISVs can affect replication of pathogenic viruses in insect cells, isolation and characterization of multiple ISVs that can co-circulate with arboviruses of medical and veterinary importance is a field of an increasing interest. Insect cell culture systems are a convenient and easy to use tool to study interactions between ISVs and arboviruses; however, they might be not fully relevant to *in vivo* conditions and cannot serve as a substitute for *in vivo* vector competence studies [6]. For example, co-infection experiments with Nhumirim virus (NHUV) (an insect-specific flavivirus) showed that C6/36 cells infected with NHUV produced 10,000-fold less infectious viral particles of West Nile virus (WNV) compared to uninfected C6/36 cells [17, 18]; however, while there were statistically significant differences in transmission rates for WNV in NHUV-infected and uninfected *Cx. quinquefasciatus* mosquitoes at day 7 and 9 post-infection, viral titer in mosquito tissues were not affected [18]. Similar observations were reported for another insect-specific flavivirus (Bamaga virus) in *Cx. annulirostris* mosquitoes [19].

## Conclusions

To our knowledge, this is the first study that evaluated the effect of AgDNV infection on MAYV replication in *An. gambiae* cells and mosquitoes. Our results support the hypothesis that ISVs can negatively modulate arbovirus infection in mosquitoes and cell cultures. Our study also highlights the importance of screening cell lines and mosquito colonies for ISVs, as these endogenous infections can potentially alter the replication of medically important arboviruses. Finally, we show that *An. gambiae*, AgDNV and MAYV can serve as a model to study the effects of ISVs on pathogenic arbovirus replication in *Anopheles* mosquitoes.

## Supplementary information

**Additional file 1: Figure S1.** Replication of AgDNV in mosquitoes 10 days post-inoculation.

## Data Availability

All data generated or analyzed during this study are included in this published article.

## Abbreviations

AgDNV: Anopheles gambiae densovirus
DMEM: Dulbecco’s Modified Eagle’s Medium
FBS: Fetal bovine serum
ISV: Insect-specific virus
GE: Genome equivalent
NHUV: Nhumirim virus
MAYV: Mayaro virus
MOI: Multiplicity of infection
PBS: Phosphate-buffered saline
WNV: West Nile virus
qPCR: Quantitative polymerase chain reaction
