# Supplementary figures and images for: Anopheles gambiae densovirus (AgDNV) negatively affects Mayaro virus infection in Anopheles gambiae cells and mosquitoes
Source: Parasit Vectors. 2020 Apr 22;13:210. doi: 10.1186/s13071-020-04072-8 (PMC7178629; doi:10.1186/s13071-020-04072-8)

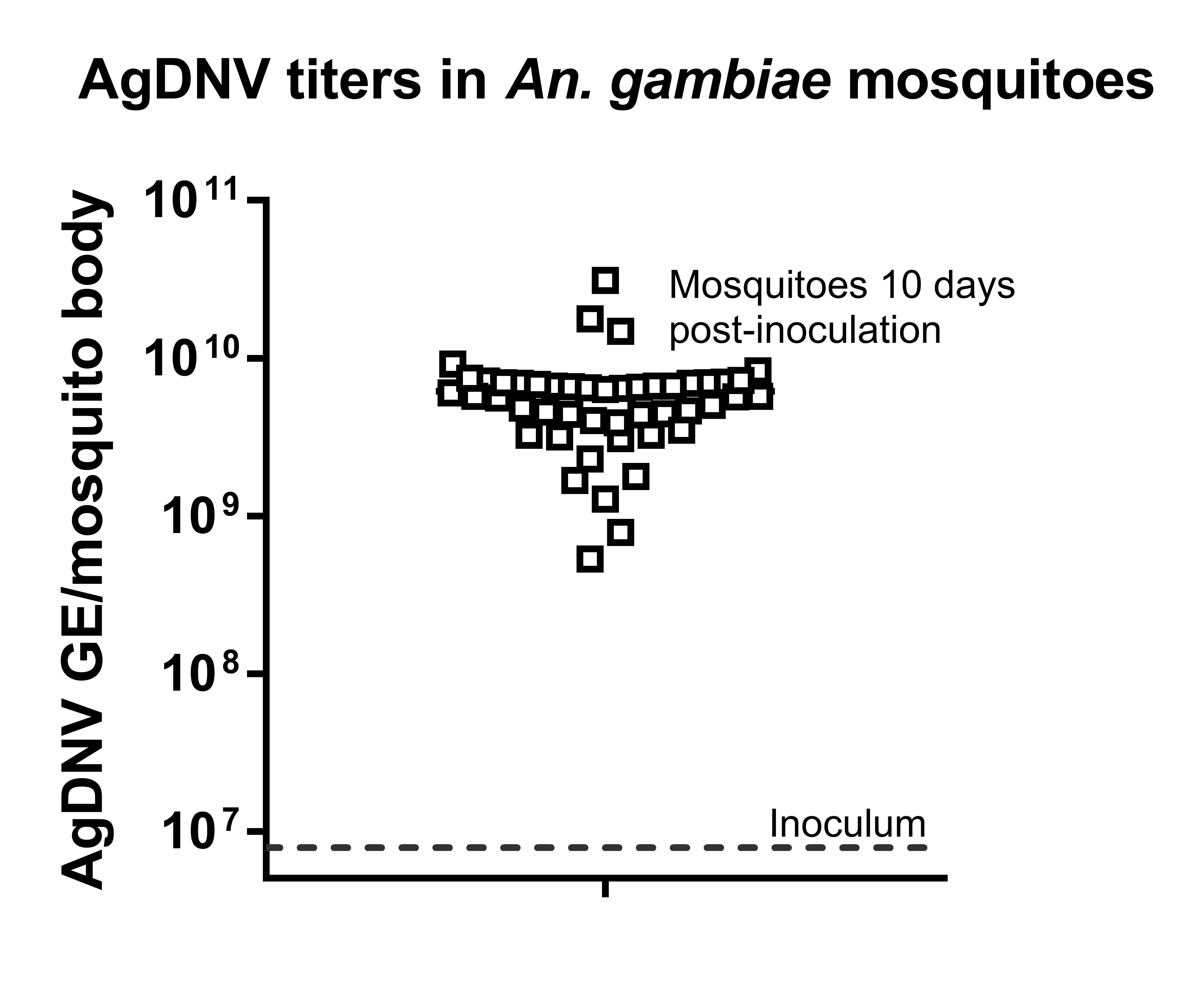

Supplement: Supplementary file 1 — Additional file 1: Figure S1. Replication of AgDNV in mosquitoes 10 days post-inoculation. [file 13071_2020_4072_MOESM1_ESM.jpg]
